# Association of Concussion Symptoms With Testosterone Levels and Erectile Dysfunction in Former Professional US-Style Football Players

**DOI:** 10.1001/jamaneurol.2019.2664

**Published:** 2019-08-26

**Authors:** Rachel Grashow, Marc G. Weisskopf, Karen K. Miller, David M. Nathan, Ross Zafonte, Frank E. Speizer, Theodore K. Courtney, Aaron Baggish, Herman A. Taylor, Alvaro Pascual-Leone, Lee M. Nadler, Andrea L. Roberts

**Affiliations:** 1 Department of Environmental Health, Harvard T. H. Chan School of Public Health, Boston, Massachusetts; 2 Football Players Health Study, Harvard Medical School, Boston, Massachusetts; 3 Environmental and Occupational Medicine and Epidemiology Program, Harvard T. H. Chan School of Public Health, Boston, Massachusetts; 4 Neuroendocrine Unit, Department of Medicine, Massachusetts General Hospital, Boston; 5 Harvard Medical School, Boston, Massachusetts; 6 Massachusetts General Hospital Diabetes Center, Boston; 7 Department of Medicine, Harvard Medical School, Boston, Massachusetts; 8 Department of Physical Medicine and Rehabilitation, Spaulding Rehabilitation Hospital, Boston, Massachusetts; 9 Massachusetts General Hospital, Harvard Medical School, Boston; 10 Brigham and Women’s Hospital, Harvard Medical School, Boston, Massachusetts; 11 Channing Division of Network Medicine, Brigham and Women’s Hospital, Harvard Medical School, Boston, Massachusetts; 12 Cardiovascular Performance Program, Massachusetts General Hospital, Boston; 13 Cardiovascular Research Institute, Morehouse School of Medicine, Atlanta, Georgia; 14 Berenson-Allen Center for Noninvasive Brain Stimulation, Division of Cognitive Neurology, Department of Neurology, Beth Israel Deaconess Medical Center, Harvard Medical School, Boston, Massachusetts; 15 Dana Farber Cancer Institute, Boston, Massachusetts

## Abstract

**Importance:**

Small studies suggest that head trauma in men may be associated with low testosterone levels and sexual dysfunction through mechanisms that likely include hypopituitarism secondary to ischemic injury and pituitary axonal tract damage. Athletes in contact sports may be at risk for pituitary insufficiencies or erectile dysfunction (ED) because of the high number of head traumas experienced during their careers. Whether multiple symptomatic concussive events are associated with later indicators of low testosterone levels and ED is unknown.

**Objective:**

To explore the associations between concussion symptom history and participant-reported indicators of low testosterone levels and ED.

**Design, Setting, and Participants:**

This cross-sectional study of former professional US-style football players was conducted in Boston, Massachusetts, from January 2015 to March 2017. Surveys on past football exposures, demographic factors, and current health conditions were sent via electronic and postal mail to participants within and outside of the United States. Analyses were conducted in Boston, Massachusetts; the data analysis began in March 2018 and additional analyses were performed through June 2019. Of the 13 720 male former players eligible to enroll who were contacted, 3506 (25.6%) responded.

**Exposures:**

Concussion symptom score was calculated by summing the frequency with which participants reported 10 symptoms, such as loss of consciousness, disorientation, nausea, memory problems, and dizziness, at the time of football-related head injury.

**Main Outcomes and Measures:**

Self-reported recommendations or prescriptions for low testosterone or ED medication served as indicators for testosterone insufficiency and ED.

**Results:**

In 3409 former players (mean [SD] age, 52.5 [14.1] years), the prevalence of indicators of low testosterone levels and ED was 18.3% and 22.7%, respectively. The odds of reporting low testosterone levels or ED indicators were elevated for previously established risk factors (eg, diabetes, sleep apnea, and mood disorders). Models adjusted for demographic characteristics, football exposures, and current health factors showed a significant monotonically increasing association of concussion symptom score with the odds of reporting the low testosterone indicator (highest vs lowest quartile, odds ratio, 2.39; 95% CI, 1.79-3.19; *P* < .001). The ED indicator showed a similar association (highest quartile vs lowest, odds ratio, 1.72; 95% CI, 1.30-2.27; *P* < .001).

**Conclusions and Relevance:**

Concussion symptoms at the time of injury among former football players were associated with current participant-reported low testosterone levels and ED indicators. These findings suggest that men with a history of head injury may benefit from discussions with their health care clinicians regarding testosterone deficiency and sexual dysfunction.

## Key Points

**Question:**

Are professional US-style football players with a history of multiple concussion symptoms more likely to report indicators of low testosterone levels or erectile dysfunction (ED)?

**Findings:**

In this cross-sectional study of 3409 former players, a monotonically increasing association was found between the number of concussion symptoms and the odds of reporting an indicator of low testosterone level and ED.

**Meaning:**

Concussion symptoms among former football players were associated with low testosterone levels and ED indicators, suggesting that men with a history of head injury may benefit from discussions with their health care clinicians regarding these treatable outcomes.

## Teaser

This cross-sectional study examines the association between self-reported concussion symptoms at the time of US-style football injury and self-reported low testosterone levels and erectile dysfunction indicators in US former professional football players.

## Introduction

Healthy sexual function is important for psychosocial well-being^1,2^ and intimate partner relations.^3,4^ *Erectile dysfunction* (ED), defined as the inability to maintain an erection sufficient for sexual activity,^5^ and pituitary hormone deficiencies may be long-term sequelae of traumatic brain injury (TBI).^6^ A plausible biological mechanism for such effects is trauma-induced pituitary damage, which may lead to insufficiencies in testosterone, growth hormone, or cortisol levels,^7,8^ termed *posttraumatic hypopituitarism*.^9,10,11^

Studies on sexual function in participants with brain injuries have reported a reduced frequency of intercourse,^10,12,13,14^ diminished libido,^10,12,13,14,15,16,17,18,19^ impaired ability to orgasm,^10,12,13,18,19^ ED,^6,10,13,14,15,17^ and sexual arousal issues.^10,12,18^ However, these studies were conducted in clinical settings,^6,9,10,12,13,14,15,16,17,18,19^ many were small (N < 100)^9,13,14,15,17,19^ or did not specifically investigate ED.^12,16,18,19^ Only 1 large study examined ED subsequent to a single TBI in 73 000 clinical patients and 218 000 controls.^6^ Over a 10-year follow-up, the adjusted hazard ratio for ED in patients with TBI was 2.5 compared with participants without injuries, and greater TBI severity was associated with higher risk of ED.^6^ However, this study focused only on medically evaluated single head injuries, rendering results less applicable to often underdiagnosed sports-related head traumas.^20,21^ Furthermore, this study did not evaluate dose-response associations with repeated head injuries and lacked covariate data, such as body mass index (BMI; calculated as weight in kilograms divided by height in meters squared).^22^

Limited research has been conducted on populations likely to receive repeated head injuries, such as athletes in combative and contact sports or the military. Small studies of professional boxers have found hormone insufficiencies^23,24,25^ and smaller pituitary volumes^23^ when compared with controls. One study of 68 National Football League (NFL) players with poor quality-of-life scores found significant associations between repeated mild head injury and pituitary and sexual dysfunction.^26^ Three small studies (all N < 40) reported that veterans with mild blast-related head injury were more likely to have a pituitary hormone insufficiency compared with civilians and uninjured veterans.^27,28,29^ Exploring these end points in professional US-style football players could yield new insights given that prior studies were small, looked only at clinically defined single head injuries, were conducted in players with a low quality of life, or were conducted in veterans with blast-related rather than mechanical trauma.

We examined the association between football-related concussion symptoms at the time of football injury and self-reported medication recommendations for low testosterone levels or ED in a large cohort of former professional US-style football players. Because former players are at increased risk for ED comorbidities, such as sleep apnea,^30,31^ cardiometabolic disease,^32,33,34^ opioid use,^35^ depression,^30,36,37,38,39,40,41^ obesity,^30^ and prior use of performance enhancing drugs,^42^ we conducted analyses further adjusted for these factors.

## Methods

### Participants

The Football Players’ Health Study (FPHS)^43^ recruited men who played for the NFL after 1960, when the adoption of hard plastic helmets was mostly complete. Of the 14 906 player addresses obtained from the NFL Players’ Association, 1186 (8.6%) were invalid. Questionnaires were sent to the remaining 13 720 former players, of whom 3506 (25.6%) had responded as of March 2017. The Beth Israel Deaconess Medical Center institutional review board approved this study. Informed written consent was obtained from all participants prior to participation.

### Concussion Symptoms

Respondents were asked: “While playing or practicing football, did you experience a blow to the head, neck, or upper body followed by any of the following: headaches, nausea, dizziness, loss of consciousness (LOC), memory problems, disorientation, confusion, seizure, visual problems, or feeling unsteady on your feet?” Response options were: none, once, 2 to 5 times, 6 to 10 times, or 11 times or more for each symptom.

### Outcomes

Respondents were asked “Has a medical provider ever recommended or prescribed medicine for: (1) low testosterone or (2) ED?” An affirmative answer served as an indicator of a history of low testosterone levels or ED, respectively. Participants reporting that a health care clinician had ever recommended or prescribed medication for either outcome were considered cases. Participants were additionally asked whether they currently took medication for low testosterone levels or ED.

### Covariates

Participants chose the category that best described their race/ethnicity and were categorized by investigators as black, white, or other. Football position may be a proxy for training regimen, nutrition, and other unmeasured variables and has been associated with injuries.^44,45,46,47^ Respondents provided the position(s) played most frequently, which included defensive back, defensive line, kicker/punter, linebacker, offensive line, quarterback, running back, special teams, tight end, or wide receiver. Respondents who selected “special teams” in addition to strength-based positions (eg, offensive line, defensive line, or tight end) were assigned “special line.”^48^ Players who selected “special teams” and speed-based positions (eg, running back, wide receiver, defensive back, or linebacker) were assigned to “special speed.” For the 1037 players (29.6% of all respondents) who endorsed multiple positions, we assigned the highest-risk position based on mild TBI risk per 100 game positions.^44^

Body mass index during professional play was calculated using self-reported height and professional weight (<25.0, 25.0-30.0, or >30.0). Participants reported the number of seasons of professional play. For 70 men (2.1%) missing these data, total seasons were calculated using the first and last year of professional play or from Pro-Football Reference (PFR) data.^49^ Participants were asked “During your active playing years, did you take any medications or other drugs to help performance?”

Participants were asked whether they had ever been recommended or prescribed medication for hypertension, high cholesterol levels, diabetes, heart failure, heart rhythm issues, and/or pain. They were separately asked if they had received a diagnosis of cancer, sleep apnea, or myocardial infarction or had undergone cardiac surgery. Participants were considered to have a heart condition if they reported heart rhythm issues, myocardial infarction, heart failure, or cardiac surgery. Self-reported weight and height were used to calculate their current BMI.

Anxious and depressive symptoms over the prior 2 weeks were assessed using the Patient Health Questionnaire 4. Responses were separately summed for anxious and depressive symptoms and dichotomized at more than 3 to indicate high depressive or anxiety symptoms.^50,51^ Participants were considered to have depression or anxiety if they reported high symptoms or were currently prescribed antidepressants or anxiolytics, respectively. Alcohol intake was quantified as the total number of alcohol beverages consumed per week.

### Statistical Analyses

We calculated the mean age, number of seasons, start year, and end year for study participants. To examine selection bias, we used PFR data to compare the FPHS cohort with all former players who played after 1960. Two-sample *t* tests and χ^2^ tests were used to identify differences between the FPHS and PFR.

Concussion symptom frequency responses of none, once, 2 to 5 times, 6 to 10 times, or 11 or more were coded as 0, 1, 3.5, 8, and 13, respectively, and then summed to create a concussion symptom score. This score was then divided into quartiles to minimize the influence of outliers. Participants who did not respond to more than 5 concussion symptoms were excluded (n = 97). Of the 3409 remaining participants, 1 or more missing symptoms were imputed for 365 players (10.7%) via multiple imputation using chained equations.^52^ Thirty-nine participants (1.1%) who did not respond to the LOC question were excluded from models examining LOC as the exposure. Data from participants who did not respond to outcome questions were excluded from related analyses (low testosterone levels, N_missing_ = 75 [2.2%]; ED, N_missing_ = 77 [2.3%]). Multiple imputation was used for covariates with missing data (N_missing_ = 3 to 88).

To determine whether indicators of low testosterone levels and ED were more prevalent among men with established low testosterone levels and ED risk factors (eg, diabetes), we examined associations between risk factors and outcomes in age- and race-adjusted models. To measure the association of concussion symptom scores with indicators of low testosterone levels and ED, we calculated odds ratios (ORs) separately for indicators of low testosterone levels and ED as the dependent variable and concussion symptoms as the independent variable after adjusting for age and race. Models were further adjusted for football exposures and current health factors in exploratory analyses. To address the possibility that recall bias may have affected the number of reported concussion symptoms, we used LOC as a more easily recalled exposure.^53,54^ To test for linear trends, the median of the concussion symptom quartile or LOC category was assigned to each participant and entered in models as a continuous variable. We additionally examined concussion scores and LOC as continuous measures. To determine whether current health factors statistically mediated associations between concussion scores and low testosterone levels or ED, we fit fully adjusted models with and without each current health factor. We calculated the percentage mediation for each variable as: 100*([β_without mediator_– β_with mediator_]/β_without mediator_).

To increase the likelihood that we were capturing men with low testosterone levels and ED, we separately considered only the subset of men who reported currently taking medication for low testosterone levels or ED as cases, excluding men who reported a history of medication recommendation or prescription but no current use. We next examined associations in younger players by restricting the data set to men 50 years or younger. We also restricted the data set to men who last played 20 years or fewer before the survey to determine whether concussion symptoms experienced 2 or more decades prior were associated with the outcomes. Depression and anxiety can lead to ED,^55,56,57^ and low testosterone levels^58^ and ED^59,60^ may cause or exacerbate depression. We therefore included indicators of depression and anxiety in sensitivity analyses. To address the possibility that stigma associated with ED was associated with the response rate, we ran analyses in which all ED nonrespondents were imputed as either “no ED” or “have ED.”

We used inverse probability weighting^61^ to account for possible selection bias from nonparticipation in the FPHS. We predicted the probability of participation in the FPHS based on position, BMI, career length, and first and last year of professional play using PFR data. The inverse of these probabilities, stabilized and truncated at the 5th and 95th percentiles to minimize the effect of outliers, were used as weights in fully adjusted models of low testosterone levels and ED.^49,61^ Odds ratios for all analyses were estimated using generalized linear models (“glm” package; R Statistical Software; R Foundation) and statistical significance was set at *P *< .05.

## Results

Table 1 shows the distribution of demographic, football, and current health-related variables by concussion symptom quartile. Participants’ mean (SD) age was 52.5 (14.1) years. Participants had played a mean (SD) of 6.8 (3.8) seasons. Respondents of the FPHS began their careers 4 years earlier than nonrespondents, ended their careers 3 years earlier, and had slightly longer careers (career start: *t* =  13.1; 95% CI, 3.2-4.4; career end: *t* = 9.3; 95% CI, 2.1-3.2; career duration: *t* = 14.3; 95% CI, −1.3 to −1.0; *P* < .001 for all). Playing position differed among respondents vs nonrespondents (offensive linemen: FPHS, 21.7%; nonrespondents, 3.6%; χ^2^ = 100.9; *P* < .001; defensive backs: FPHS, 14.8%; nonrespondents, 9.9%; χ^2^ = 40.7; *P* < .001; running backs: FPHS, 9.4%; nonrespondents, 13.3%; χ^2^ = 32.3; *P* < .001; tight ends: FPHS, 7.7%; nonrespondents, 5.9%; χ^2^ = 11.7; *P* < .001; wide receivers: FPHS, 0.5%; nonrespondents, 12.4%; χ^2^ = 7.9; *P* < .001).

**Table 1.** Demographic, Football, and Current Health Factors by Concussion Symptom Quartile for 3409 Participants

Quartile (Concussion Score Range)	Concussion Symptom Quartile, No. (%)			
	1 (0.0-10.5)	2 (10.5-23.0)	3 (23.5-43.5)	4 (43.5-130.0)
No.	853 (25.0)	852 (25.0)	852 (25.0)	852 (25.0)
**Demographic Factors**				
Age, y				
21-40	202 (23.7)	198 (23.2)	228 (26.8)	240 (28.2)
41-60	308 (36.1)	341 (40.0)	398 (46.7)	416 (48.8)
>60	343 (40.2)	313 (36.7)	226 (26.5)	196 (23.0)
Race/ethnicity				
Black	310 (36.6)	286 (34.2)	347 (41.2)	331 (39.1)
White	514 (60.8)	534 (63.9)	479 (56.8)	475 (56.1)
Other	22 (2.6)	16 (1.9)	17 (2.0)	40 (4.7)
**Football Exposures**				
BMI while playing professional football^a^				
<25.0	63 (7.4)	36 (4.2)	49 (5.8)	39 (4.6)
25.0-30.0	427 (50.1)	404 (47.4)	379 (44.5)	322 (37.8)
>30.0	363 (42.6)	412 (48.4)	423 (49.6)	491 (57.6)
Professional use of PED	87 (10.2)	99 (11.6)	135 (15.8)	229 (26.9)
Position				
Defensive back	117 (13.7)	118 (13.8)	142 (16.7)	122 (14.3)
Defensive line	104 (12.2)	83 (9.7)	82 (9.6)	100 (11.7)
Kicker/punter	61 (7.2)	23 (2.7)	14 (1.6)	6 (0.7)
Linebacker	81 (9.5)	79 (9.3)	86 (10.1)	113 (13.3)
Offensive line	137 (16.1)	179 (21.0)	155 (18.2)	157 (18.4)
Quarterback	51 (6.0)	58 (6.8)	36 (4.2)	18 (2.1)
Running back	70 (8.2)	68 (8.0)	87 (10.2)	94 (11.0)
Special teams only	3 (0.4)	10 (1.2)	8 (0.9)	6 (0.7)
Special speed	20 (2.3)	34 (4.0)	43 (5.0)	37 (4.3)
Special strength	36 (4.2)	49 (5.8)	48 (5.6)	56 (6.6)
Tight end	59 (6.9)	64 (7.5)	68 (8.0)	71 (8.3)
Wide receiver	114 (13.4)	87 (10.2)	83 (9.7)	72 (8.5)
Current health-related factors				
Hypertension	322 (37.7)	306 (35.9)	326 (38.3)	329 (38.6)
High cholesterol levels	272 (31.9)	289 (33.9)	309 (36.3)	303 (35.6)
Diabetes	60 (7.0)	92 (10.8)	76 (8.9)	72 (8.5)
Heart condition^b^	160 (18.8)	170 (20.0)	155 (18.2)	152 (17.8)
Prescription pain medication	127 (14.9)	203 (23.8)	280 (32.9)	360 (42.3)
Prostate or testicular cancer	33 (3.9)	42 (4.9)	24 (2.8)	33 (3.9)
Sleep apnea	127 (14.9)	178 (20.9)	198 (23.2)	257 (30.2)
Current BMI^a^				
<25.0	60 (7.0)	46 (5.4)	52 (6.1)	20 (2.3)
25.0-30.0	388 (45.5)	372 (43.7)	337 (39.6)	333 (39.1)
>30.0	401 (47.0)	429 (50.4)	457 (53.6)	494 (58.0)
Mood indicators				
Anxiety only	28 (3.3)	42 (4.9)	68 (8.0)	98 (11.5)
Depression only	17 (2.0)	30 (3.5)	47 (5.5)	45 (5.3)
Depression and anxiety	26 (3.0)	68 (8.0)	113 (13.3)	262 (30.8)
Alcohol drinks per wk				
None	268 (31.4)	270 (31.7)	253 (29.7)	271 (31.8)
1-7	314 (36.8)	329 (38.6)	309 (36.3)	314 (36.9)
8-14	158 (18.5)	146 (17.1)	145 (17.0)	137 (16.1)
≥15	99 (11.6)	101 (11.9)	134 (15.7)	122 (14.3)

Abbreviations: BMI, body mass index; PED, performance-enhancing drugs.

^a^ Calculated as weight in kilograms divided by height in meters squared.

^b^ Heart condition includes self-reported heart rhythm issues, myocardial infarction, heart failure, or cardiac surgery.

Of 3409 participants, 611 (18.3%) reported indicators of low testosterone levels, and 755 (22.7%) reported indicators of ED. Fewer than 10% of participants reported indicators of low testosterone levels and ED (335 [9.8%]). Of 611 players with low testosterone indicators, 243 (39.8%) were currently taking medication. Among players with indicators of ED, half were currently taking medication (379 [50.2%]). The prevalence of indicators of low testosterone levels and ED was greater in men with established risk factors (Table 2). In models that included age and race, indicators of low testosterone levels and ED were significantly associated with hypertension, high cholesterol levels, diabetes, heart conditions, prescription pain medication, reproductive cancer, sleep apnea, obesity, and mood disorders (Table 3).

**Table 2.** Prevalence of History of Low Testosterone Levels and Erectile Dysfunction Indicators by Demographic, Football, and Current Health Factors for 3409 Participants

Characteristic	No.	Prevalence of History of Prescription Recommendation by Self-report, No. (%)	
		Low Testosterone Levels	Erectile Dysfunction
All		611 (18.3)	755 (22.7)
**Demographic Factors**			
Age, y			
21-40	868	70 (8.2)	46 (5.4)
41-60	1463	301 (20.9)	307 (21.4)
>60	1078	240 (23.1)	402 (38.6)
Race			
Black	1274	210 (16.8)	280 (22.5)
White	2002	373 (19.1)	454 (23.2)
Other	95	23 (24.2)	14 (15.2)
Missing	38	5 (13.5)	7 (18.9)
**Football Exposures**			
BMI while playing professional football^a^			
<25.0	187	33 (17.7)	36 (19.9)
25.0-30.0	1532	243 (16.3)	353 (23.6)
>30.0	1689	334 (20.2)	365 (22.0)
Professional use of PED			
No	2859	471 (16.8)	608 (21.8)
Yes	550	140 (26.1)	147 (27.3)
**Current Health-Related Factors**			
Hypertension			
No	2083	287 (13.9)	297 (14.5)
Yes	1283	318 (25.5)	450 (35.9)
High cholesterol levels			
No	2165	292 (13.7)	352 (16.5)
Yes	1173	309 (27.0)	387 (33.6)
Diabetes			
No	3021	504 (16.9)	596 (20.0)
Yes	300	95 (32.5)	136 (46.7)
Heart condition^b^			
No	2772	437 (16.0)	522 (19.2)
Yes	637	174 (28.5)	233 (37.8)
Prescription pain medication			
No	2439	328 (13.7)	415 (17.4)
Yes	970	283 (29.9)	340 (35.7)
Prostate or testicular cancer			
No	3277	576 (18)	686 (21.4)
Yes	132	35 (27.1)	69 (54.3)
Sleep apnea			
No	2582	367 (14.5)	478 (18.8)
Yes	760	236 (31.9)	264 (35.7)
Current BMI^a^			
<25.0	178	16 (9.1)	32 (18.4)
25.0-30.0	1430	211 (15.1)	276 (19.8)
>30.0	1781	383 (22.0)	442 (25.4)
Mood indicators^c^			
No depression or anxiety	2562	357 (14.3)	479 (19.1)
Anxiety only	236	44 (19.0)	49 (21.2)
Depression only	139	36 (26.5)	43 (31.9)
Depression and anxiety	469	174 (37.8)	184 (40)
Alcohol drinks per wk			
None	1062	213 (20.5)	240 (23.1)
1-7	1266	210 (17.0)	266 (21.5)
8-14	586	97 (17.0)	128 (22.1)
≥15	456	85 (19.0)	111 (24.8)

Abbreviations: BMI, body mass index; PED, performance-enhancing drugs.

^a^ Calculated as weight in kilograms divided by height in meters squared.

^b^ Heart condition includes self-reported heart rhythm issues, myocardial infarction, heart failure, or cardiac surgery.

^c^ Derived from Patient Health Questionnaire 4.

**Table 3.** Low Testosterone Levels or ED Indicators in Association With Established Low Testosterone Levels and ED Risk Factors for 3409 Participants

Characteristic	Prevalence of History of Prescription Recommendation by Self-report, OR (95% CI)	
	Low Testosterone	ED
**Model 1: Mutually Adjusted for Age and Race**		
Age, y		
21-40	1 [Reference]	1 [Reference]
41-60	2.99 (2.27-3.94)^a^	4.82 (3.49-6.66)^a^
>60	3.41 (2.56-4.55)^a^	12.22 (8.79-16.98)^a^
Race/ethnicity		
White	1 [Reference]	1 [Reference]
Black	0.97 (0.80-1.17)	1.4 (1.17-1.69)^a^
Other	1.61 (0.98-2.64)	0.87 (0.47-1.59)
Missing	0.64 (0.25-1.67)	0.63 (0.27-1.50)
**Models 2-10: Age and Race Adjusted**		
Model 2: hypertension	1.81 (1.50-2.19)^a^	2.26 (1.89-2.71)^a^
Model 3: high cholesterol levels	1.96 (1.62-2.37)^a^	1.69 (1.41-2.02)^a^
Model 4: diabetes	2.04 (1.55-2.69)^a^	2.66 (2.04-3.45)^a^
Model 5: heart condition^b^	1.73 (1.39-2.15)^a^	1.64 (1.34-2.01)^a^
Model 6: prescription pain medication	2.53 (2.10-3.05)^a^	2.3 (1.93-2.75)^a^
Model 7: prostate or testicular cancer	1.31 (0.87-1.97)	2.54 (1.75-3.69)^a^
Model 8: sleep apnea	2.51 (2.07-3.05)^a^	2.04 (1.69-2.46)^a^
Model 9: current BMI^c^		
<25.0	1 [Reference]	1 [Reference]
25.0-30.0	1.93 (1.13-3.32)^d^	1.35 (0.88-2.06)
>30.0	3.11 (1.82-5.30)^a^	1.99 (1.31-3.02)^e^
Model 10: mood disorders		
None	1 [Reference]	1 [Reference]
Anxiety indicators only	1.63 (1.14-2.33)^e^	1.59 (1.12-2.26)^d^
Depression indicators only	2.22 (1.48-3.34)^a^	2.11 (1.41-3.15)^a^
Depression and anxiety indicators	4 (3.19-5.02)^a^	3.37 (2.67-4.24)^a^
Model 11: alcohol drinks per wk		
None	1 [Reference]	1 [Reference]
1-7	0.84 (0.68-1.05)	1.03 (0.84-1.27)
8-14	0.81 (0.61-1.06)	0.99 (0.76-1.28)
15+	0.94 (0.71-1.26)	1.27 (0.96-1.67)

Abbreviations: BMI, body mass index; ED, erectile dysfunction; OR, odds ratio.

^a^ *P* < .001.

^b^ Heart condition includes self-reported heart rhythm issues, myocardial infarction, heart failure, or cardiac surgery.

^c^ Calculated as weight in kilograms divided by height in meters squared.

^d^ *P* < .05.

^e^ *P* < .01.

We found statistically significant, monotonically increasing associations between concussion symptoms and indicators for low testosterone levels and ED in models adjusted for age and race (Figure; low testosterone OR, 3.49; 95% CI, 2.68-4.56; ED OR, 2.41; 95% CI, 1.87-3.11). In models further adjusted for professional football–related exposures (eg, position, BMI during professional play, and self-reported use of performance enhancing drugs), estimates remained essentially unchanged from age- and race-adjusted models (Figure; low testosterone OR, 3.38; 95% CI, 2.57-4.45; ED OR, 2.32; 95% CI, 1.78-3.02).

**Figure.**
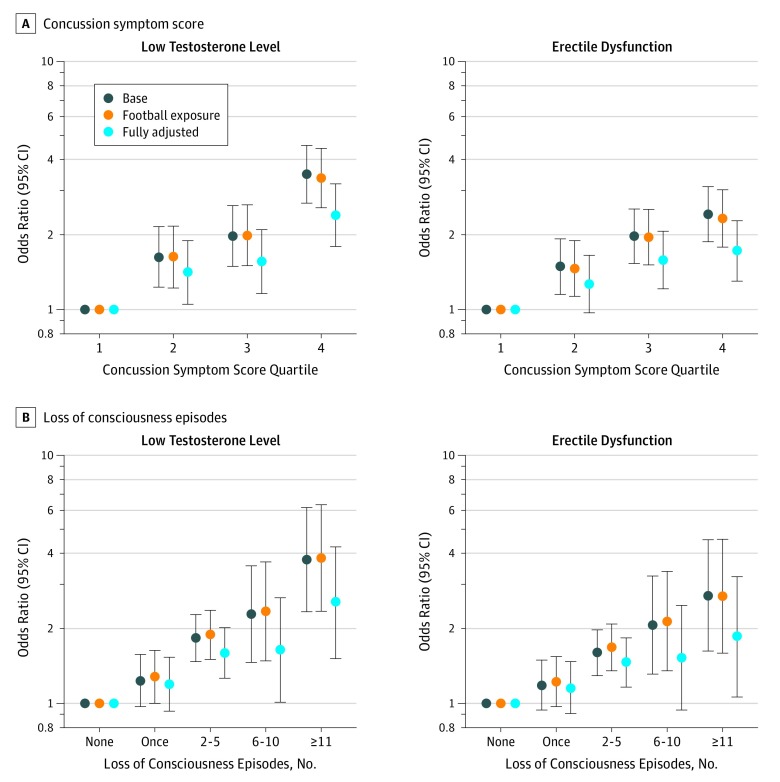
Association Between Concussion Symptom Quartile and Loss of Consciousness With Low Testosterone and Erectile Dysfunction A and B, The lowest quartile served as the reference for all models. The base model is adjusted for age and race/ethnicity; the football exposure model is further adjusted for body mass index (BMI) (calculated as weight in kilograms divided by height in meters squared) while playing professional football, position, and use of performance-enhancing drugs; and the fully adjusted model is further adjusted for current BMI, heart condition, diabetes, high cholesterol levels, hypertension, sleep apnea, use of prescription pain medication, alcohol drinks per week, and a history of testicular or prostate cancer. Linear tests of trend were significant (*P *< .01).

Associations in models further adjusted for current health factors were slightly attenuated but remained statistically significant (Figure; low testosterone OR, 2.39; 95% CI, 1.79-3.19; ED OR, 1.72; 95% CI, 1.30-2.27). Loss of consciousness was associated with indicators of low testosterone levels and ED in models adjusted for demographics, football exposures, and current risk factors (Figure). In fully adjusted models with concussion symptoms and LOC coded as continuous variables, both were significantly associated with low testosterone levels and ED (concussion symptoms: low testosterone β = 1.01; 95% CI, 1.01-1.02; *P* ≤ .001; ED β = 1.01; 95% CI, 1.01-1.01; *P* ≤ .001; LOC: low testosterone β = 1.08; 95% CI, 1.04-1.12; *P* ≤ .001; ED β = 1.06; 95% CI, 1.02-1.10; *P* = .001). For low testosterone, men with relatively low concussion scores (the second quartile) had significantly elevated ORs compared with men in the lowest quartile (OR, 1.41; 95% CI, 1.05-1.89; *P* = .02).

The largest statistical mediators of the association between concussion score and the outcomes were current use of prescription pain medication (low testosterone mediation, 7.9%; ED mediation, 20.4%) and sleep apnea (low testosterone mediation, 9.7%; ED mediation, 5.9%). All other current health factors statistically mediated the associations by less than 4%.

Results were similar in analyses restricted to participants currently using low testosterone and ED medication among players younger than 50 years, among players who played 20 years or longer before the study, and in inverse probability–weighted analyses. Further adjustment for mood indicators somewhat attenuated associations (Table 4). We conducted a post hoc analysis to compare associations among men with low testosterone levels only, ED only, and men with both. Associations with concussion symptoms were stronger among men reporting low testosterone levels and ED compared with men reporting only 1 of the 2 outcomes (eTable in the Supplement; highest concussion quartile vs lowest; men with low testosterone only: OR, 2.66; 95% CI, 1.84-3.83, *P* < .001; men with ED only: OR, 1.47; 95% CI, 1.06-2.04; *P* = .02; men with both outcomes: OR, 4.95; 95% CI, 3.40-7.22; *P* < .001).

**Table 4.** Sensitivity Analyses for Low Testosterone Levels and Erectile Dysfunction Indicators for Each Quartile of Concussion Symptom Score for 3409 Participants

Model	No.		Concussion Symptom Quartile	History of Prescription Recommendation, OR (95%)	
	Low Testosterone Levels	Erectile Dysfunction		Low Testosterone Levels	Erectile Dysfunction
Model 1: base model^a^	3334	3332	1	1 [Reference]	1 [Reference]
			2	1.62 (1.23-2.15)^b^	1.49 (1.15-1.92)^b^
			3	1.97 (1.49-2.6)^c^	1.96 (1.53-2.53)^c^
			4	3.49 (2.68-4.56)^c^	2.41 (1.87-3.11)^c^
Model 2: case definition includes only current medication users^a^	3334	3332	1	1 [Reference]	1 [Reference]
			2	1.67 (1.10-2.56)^d^	1.21 (0.88-1.67)
			3	1.93 (1.26-2.94)^b^	1.65 (1.21-2.24)^b^
			4	3.02 (2.02-4.5)^c^	1.62 (1.18-2.24)^b^
Model 3: restricted to men ≤50 y^a^	1460	1457	1	1 [Reference]	1 [Reference]
			2	1.41 (0.84-2.38)	1.69 (0.90-3.18)
			3	1.72 (1.05-2.83)^d^	2.75 (1.53-4.93)^b^
			4	2.92 (1.83-4.66)^c^	3.29 (1.85-5.85)^c^
Model 4: men who last played ≥20 y prior^a^	1953	1953	1	1 [Reference]	1 [Reference]
			2	1.57 (1.14-2.17)^b^	1.48 (1.12-1.95)^b^
			3	1.76 (1.27-2.44)^b^	1.79 (1.35-2.38)^c^
			4	3.08 (2.24-4.24)^c^	2.09 (1.56-2.80)^c^
Model 5: fully adjusted including mood disorders^e^	3334	3332	1	1 [Reference]	1 [Reference]
			2	1.33 (0.99-1.78)	1.19 (0.91-1.56)
			3	1.41 (1.05-1. 90)^d^	1.43 (1.09-1.88)^d^
			4	1.89 (1.39-2.55)^c^	1.36 (1.02-1.83)^d^
Model 6: missing imputed as no^a^	3409	3409	1	1 [Reference]	1 [Reference]
			2	1.61 (1.22-2.13)^b^	1.48 (1.15-1.91)^b^
			3	1.91 (1.45-2.52)^c^	1.96 (1.52-2.52)^c^
			4	3.43 (2.63-4.48)^c^	2.34 (1.82-3.02)^c^
Model 7: missing imputed as yes^a^	3409	3409	1	1 [Reference]	1 [Reference]
			2	1.67 (1.29-2.17)^c^	1.51 (1.18-1.92)^b^
			3	2 (1.54-2.60)^c^	1.93 (1.52-2.46)^c^
			4	3.37 (2.61-4.34)^c^	2.46 (1.92-3.14)^c^
Model 8: inverse probability weighted^e^	3334	3332	1	1 [Reference]	1 [Reference]
			2	1.40 (1.04-1.89)^d^	1.34 (1.01-1.77)^d^
			3	1.50 (1.11-2.03)^b^	1.65 (1.25-2.19)^c^
			4	2.44 (1.82-3.29)^c^	1.90 (1.43-2.54)^c^

Abbreviation: OR, odds ratio.

^a^ Adjusted for age and race.

^b^ *P* < .01.

^c^ *P* < .001.

^d^ *P* < .05.

^e^ Adjusted for age, race, professional body mass index (calculated as weight in kilograms divided by height in meters squared), position, use of performance-enhancing drugs, current body mass index, heart condition (eg, heart rhythm issues, myocardial infarction, heart failure, or cardiac surgery), diabetes, high cholesterol levels, hypertension, sleep apnea, alcohol beverages per week, use of prescription pain medication, and history of testicular or prostate cancer.

## Discussion

We identified a highly robust, monotonically increasing association between self-reported concussion symptoms at the time of football injury and self-reported low testosterone levels and ED indicators. Even participants with relatively few concussion symptoms (ie, those in the second quartile) had significantly elevated odds of reporting low testosterone levels compared with men in the lowest quartile.

Our findings add to a literature composed of studies linking single head injuries with pituitary dysfunction in the general population,^6,9,10,11^ small studies of professional boxers,^23,24,25,26,62,63^ and findings from veterans with blast-induced head injury,^27,28,29^ indicating that mechanical and blast-induced trauma may have associations with pituitary and sexual function. To our knowledge, this is the first large study to examine low testosterone levels and ED, albeit indirectly, in a nonclinical population with a high prevalence of repeated injuries. This is also the first study to adjust for risk factors such as BMI.

Several hypothesized mechanisms, including concussion-associated hypopituitarism, may explain the associations of concussion with low testosterone levels and ED. The pituitary gland is perfused by long portal vessels branching off the internal carotid artery,^64^ making it susceptible to mechanical trauma, low cerebral blood flow, and increased intracranial pressure associated with head injury.^65,66^ Acceleration and deceleration forces can shear axonal tracts that connect the pituitary to the hypothalamus. Thus, the combination of intracranial pressure, reduced blood flow, and diffuse axonal injury between the pituitary and the hypothalamus could cause diminished pituitary function, leading to low testosterone levels and ED. In exploratory mediation analyses, we found that adjusting for current prescription pain medication use and sleep apnea modestly attenuated the association of concussion symptoms with low testosterone levels and ED. These results suggest that pain medication and sleep apnea should be explored as possible pathways through which head injury affects hormone levels and sexual function.

### Limitations

Our study has several limitations. First, we used indirect measures of low testosterone levels and ED. However, participant-reported health care clinician medication recommendations may be reasonable proxies: self-reported sexual dysfunction single-question assessments^67,68^ have largely replaced invasive physiological tests in clinical and research settings.^69,70,71^ Medical records, often a criterion standard for case ascertainment for other outcomes, may be less reliable for sexual dysfunction given that only 30% of men seek medical treatment for ED.^72^ Nevertheless, US men are comparatively more likely to seek treatment vs men in European countries (56% vs 10%-47%) and more willing to take ED medication.^73^ Moreover, the validity of our indicators is supported by statistically significant associations with known low testosterone and ED risk factors and by sensitivity analyses in men who self-reported currently using testosterone therapy and ED medication.

Second, concussion data were collected retrospectively, raising the possibility of recall bias. However, the robust monotonic association of the concussion exposure with the outcomes suggests simple recall bias may not solely account for our findings. Third, the concussion symptom scale has not been validated. To our knowledge, there is no validated retrospective measure of concussion symptoms. In our data, findings were similar using LOC count.^53,54,74,75^ Moreover, simpler metrics, such as the number of diagnosed concussions, may not adequately capture the experience of concussion or concussion severity; at least 30% of concussions may be undiagnosed,^21,75,76^ players may hide concussions,^21^ and concussion management during professional play has changed over time.^45^ Concussion symptoms have been used previously as a surrogate for head injury exposure and severity.^77,78,79,80^

Fourth, we do not know whether low testosterone levels or ED preceded men’s exposure to professional football. Fifth, the stigma surrounding sexual dysfunction could affect participants’ likelihood of speaking to their health care clinician or responding honestly on the survey.^81^ However, this would only produce the results presented in this article if such under-reporting were less likely among men with more reported concussion symptoms. Sixth, bias from the relatively low participation rate could have affected our estimates,^82^ although statistically significant monotonic relationships persisted in inverse probability of participation–weighted analyses. Seventh, illicit drug use may affect low testosterone^83,84^ and ED^85^; however, we did not query illicit drug use. Finally, health status may have been associated with players’ decisions to participate: the healthiest players may have been less motivated to participate and the players with the most impairment may have been unable to participate.^82^ However, measures of association would be biased only if participation was concurrently associated with the exposure (concussion symptoms) and the outcome (low testosterone levels or ED).^86^

## Conclusions

This study’s data suggest that concussion symptoms experienced during playing years may place NFL players at risk of low testosterone levels and ED decades later. These findings have implications for civilians and veterans who have experienced head injury, as well as for participants in combative and contact sports (eg, mixed martial arts, hockey, boxing, and soccer) who may experience repeated head trauma. Replication of our findings among nonprofessional football players and in the general population is a critical next step. Treatments for testosterone insufficiency and ED, including testosterone replacement therapy and phosphodiesterase type 5 inhibitors, are generally considered safe and have high efficacy rates.^87,88,89^ Our results could encourage clinicians to proactively query these treatable outcomes in patients with brain injuries as well as motivate future longitudinal studies to increase our understanding of the causal association between concussion and low testosterone levels and ED.

## Notes

Supplement. eTable**. **Odds ratios and 95% confidence intervals (CI) for players reporting low testosterone indicator only (Model 1), players reporting ED indicator only (Model 2), or players reporting both low testosterone and ED indicators (Model 3) compared to players reporting neither outcome. All models are age- and race-adjusted. Results are shown for each quartile of concussion symptom score where the lowest quartile serves as the reference group.
