# Supplementary material for: Association of Concussion Symptoms With Testosterone Levels and Erectile Dysfunction in Former Professional US-Style Football Players
Source: JAMA Neurol. 2019 Aug 26;76(12):1428–38. doi: 10.1001/jamaneurol.2019.2664 (PMC6714010; doi:10.1001/jamaneurol.2019.2664)
Supplement: Supplement. — eTable. Odds ratios and 95% confidence intervals (CI) for players reporting low testosterone indicator only (Model 1), players reporting ED indicator only (Model 2), or players reporting both low testosterone and ED indicators (Model 3) compared to players reporting neither outcome. All models are age- and race-adjusted. Results are shown for each quartile of concussion symptom score where the lowest quartile serves as the reference group. [file jamaneurol-76-1428-s001.pdf]

Supplementary Online Content

Grashow R, Weisskopf MG, Miller KK, et al. Association of concussion symptoms with testosterone levels and erectile dysfunction in former professional US-style football players. *JAMA Neurol*. Published online August 26, 2019. doi:10.1001/jamaneurol.2019.2664

eTable. Odds ratios and 95% confidence intervals (CI) for players reporting low testosterone indicator only (Model 1), players reporting ED indicator only (Model 2), or players reporting both low testosterone and ED indicators (Model 3) compared to players reporting neither outcome. All models are age- and race-adjusted. Results are shown for each quartile of concussion symptom score where the lowest quartile serves as the reference group.

This supplementary material has been provided by the authors to give readers additional information about their work.

© Grashow R et al. *JAMA Neurology*.

eTable. Odds ratios and 95% confidence intervals (CI) for players reporting low testosterone indicator only (Model 1), players reporting ED indicator only (Model 2), or players reporting both low testosterone and ED indicators (Model 3) compared to players reporting neither outcome. All models are age- and race-adjusted. Results are shown for each quartile of concussion symptom score where the lowest quartile serves as the reference group.

**Model**	**Concussion symptom quartile**	**OR (95% CI)**
**Model 1: Players reporting history of low testosterone medication recommendation vs. players with no history of either condition (N = 2679, N_{cases} = 268).**		
	1	Reference
	2	1.37 (0.92, 2.02)
	3	1.46 (0.99, 2.16)
	4	2.66 (1.84, 3.83)***
**Model 2: Players reporting history of ED med recommendation vs. players with no history of either condition (N= 2806, N_{cases} =395).**		
	1	Reference
	2	1.26 (0.93, 1.71)
	3	1.45 (1.07, 1.98)*
	4	1.47 (1.06, 2.04)*
**Model 3: Players reporting history of ED AND LT med recommendation vs. players with no history of either condition (N= 2746, N_{cases} =335).**		
	1	Reference
	2	2.21 (1.49, 3.26)***
	3	2.92 (1.99, 4.30)***
	4	4.95 (3.40, 7.22)***

* p<0.05; ** p<0.01, ***p<0.001.

© Grashow R et al. *JAMA Neurology*.
